# Henry Hobart Keech

**Published:** 1884-04

**Authors:** 


### IN MEMORIAM.

HENRY HOBART KEECH, M. D., D. D. S., died April 3d, 1884, of pheumonia, after a brief illness, at his residence in Baltimore City, at the age of fifty-three years. Dr. Keech graduated at the Baltimore College of Dental Surgery, in the class of 1857, and immediately afterwards began the practice of dentistry in Baltimore, succeeding Dr. Henry Snowden.

Through Dr. Snowden's influence, Dr. Keech, some years after he began practice, was appointed to the position of Demonstrator of Operative Dentistry in the Baltimore College, which he acceptably held for several years, when his rapidly increasing practice compelled him to resign. He received the degree of Doctor of Medicine from the "Washington University," which has now ceased to exist. He was also a permanent member of St. Barnabas P. E Church, and with others was instrumental in the establishment of a mission in the eastern suburbs of the city, which is now known as the "Church of the Holy Evangelists," to which he gave much attention in the endeavor to do all in his power as a sincere and consistent Christian. His dental practice was large and lucrative, and he enjoyed the utmost confidence and respect of his patients, being truly conscientious in all his duties.

The great problem of life is not simply to know oneself, but to know our fellow-men, to learn to love them, and with them labor for each other's good; not only the good which this life vouchsafes to all who honestly seek it, but that greater good which shall make us to share in the riches to follow a life well spent for the good of man and the glory of God. Such a man was Dr. Henry H. Keech, who, having faithfully and acceptably discharged his duty in an honored trust, has left a noble record behind—an honest man and a sincere Christian gentleman.

What better or more deserving tribute could be paid to any one? Esteemed for his good qualities, his affable and equable character in public, he was even more loved in his private circle for his tender and affectionate nature, which beamed through all his labors for the welfare of others. The loss of such a one, in whose construction so happy a combination of the nobler qualities of heart, mind and soul existed, is beyond the power of measurement. With the frail and aching body laid to its quiet repose, the deathless treasure of his loving heart and ready mind are left to those who were dearest to him, and so he yet remains

"Alive to us,
Who, through the lapse of years, will mark,
The after-glow his sunset, luminous,
Throws back upon our path."

"Man dies but his memory lives."

F. J. S. G.

